# Global, regional, and national temporal trends in mortality and disease burden of nasopharyngeal carcinoma attributable to smoking from 1990 to 2021 and predictions to 2040

**DOI:** 10.18332/tid/204742

**Published:** 2025-06-30

**Authors:** Defeng Liu, Lulu Zhuang, Yueze Li, Jinming Yu, Minghuan Li

**Affiliations:** 1 Cheeloo College of Medicine, Shandong University, Jinan, China; 2 Department of Radiation Oncology, Shandong Cancer Hospital, Cheeloo College of Medicine, Shandong University, Jinan, China; 3 Department of Radiation Oncology, Shandong Cancer Hospital and Institute, Shandong First Medical University and Shandong Academy of Medical Sciences, Jinan, China

**Keywords:** smoking, nasopharyngeal carcinoma, death, disease burden, epidemiology

## Abstract

**INTRODUCTION:**

Smoking is a major environmental risk factor for nasopharyngeal carcinoma (NPC), but the global burden and epidemiological trends of NPC attributable to smoking remain unclear.

**METHODS:**

Data were obtained from the 2021 Global Burden of Disease study. A comprehensive analysis was conducted on mortality, years lived with disability (YLDs), years of life lost (YLLs), disability-adjusted life years (DALYs) attributable to NPC attributable to smoking. Clustering analysis was applied to evaluate the variation patterns across 21 regions. The NORDPRED age-period-cohort model was used for prediction.

**RESULTS:**

In 2021, there were 13410 deaths globally from NPC attributable to smoking, 10031 YLDs, 1379583 YLLs, and 389614 DALYs. The disease burden was most severe in Asia. Males bore a significantly higher burden than females, mainly concentrated in middle-aged and older populations. From 1990 to 2021, although the number of cases increased, ASRs showed a marked decline, particularly among females and in regions with both high and low sociodemographic index (SDI) levels. Regional analyses revealed significant reductions of the disease burden in Australasia and Western Europe. The 21 regions were divided into 4 groups based on changes in mortality, representing distinct variation patterns. Projections from 2022 to 2040 indicate that, while the total number of deaths and disease burden is expected to rise, ASRs are anticipated to decline except YLDs.

**CONCLUSIONS:**

Smoking contributes significantly to the disease burden of NPC, posing a serious threat to public health. Targeted intervention strategies should be implemented according to the regional clustering results of disease burden.

## INTRODUCTION

Nasopharyngeal carcinoma (NPC) is a malignant tumor originating from the epithelial tissues of the nasopharynx. According to global cancer statistics, the estimated global incidence of NPC in 2022 was 120416 cases, with 73476 deaths, ranking it 23rd in incidence and 21st in cancer-related mortality worldwide^1^. Although advances in combined treatment modalities, including radiotherapy, chemotherapy, and immunotherapy, have significantly improved the prognosis of NPC patients^2^, the disease remains challenging due to its insidious onset. Approximately 70% of patients are diagnosed at a locally advanced stage, with the 5-year survival rate dropping from 94.0% in stages I-II to 73.7% in stage IV^3,4^. Furthermore, NPC incidence and mortality exhibit notable geographical and demographic variations. Asia accounts for 83.3% of the global NPC incidence and 83.6% of related deaths^1^. The highest incidence is observed in China, where there is a gradient of increasing disease prevalence from north to south^5^. In contrast, North America and Europe are non-endemic regions with low NPC prevalence. Over the past two decades, the global incidence of NPC has shown an upward trend, with an estimated annual percentage change (EAPC) of 1.59%, while the age-standardized death rate (ASDR) has declined, with an EAPC of -0.63%^6^. These incidence and mortality trends may be influenced by multiple factors, including socioeconomic conditions.

Among environmental factors, smoking has been conclusively linked to NPC development^7^. This association is most evident in the differentiated histological subtype, which paradoxically constitutes the majority of NPC cases in populations with generally low disease prevalence^8,9^. Evidence indicates that the risk of developing NPC escalates with greater smoking intensity, longer duration, higher cumulative exposure, and younger age at smoking initiation^10,11^. Prospective and retrospective studies show that smoking increases NPC risk and is strongly linked to disease progression and prognosis^12,13^. Global tobacco surveillance data indicate that approximately 1.1 billion adults worldwide are smokers, with 80% residing in low- and middle-income countries^14,15^. Despite the implementation of strict tobacco control measures in many countries in recent years, smoking rates continue to rise in certain regions, especially among younger populations^16^. This trend could have a significant impact on the future burden of NPC.

Currently, research on the mortality burden of NPC attributable to smoking is relatively limited. Most studies focus on specific regions or populations^17,18^, lacking a systematic global assessment. The Global Burden of Disease (GBD) study provides a valuable platform for analyzing the smoking attributed mortality burden of NPC. Covering 204 countries and territories, the GBD study employs standardized methods to assess disease burden, providing essential insights into the spatial and temporal distribution and trends.

Therefore, this study aims to conduct a comprehensive analysis of mortality rates, years lived with disability (YLDs), years of life lost (YLLs), disability-adjusted life years (DALYs), and the EAPC for NPC attributable to smoking from 1990 to 2021 based on GBD 2021 data. Additionally, this study forecasts the disease burden from 2022 to 2040. By analyzing the burden characteristics across different regions, genders, and socioeconomic levels, this study provides scientific evidence to guide targeted prevention and control strategies.

## METHODS

### Data source

Data for this study were sourced from the GBD 2021. The GBD 2021 provides accessible epidemiological assessments of 371 diseases and injuries, along with 88 risk factors, across 21 GBD regions and 204 countries/territories from 1990 to 2021^19^. Detailed descriptions of these data sources and their validation processes are systematically available through the Global Health Data Exchange (GHDx) tool (http://ghdx.healthdata.org/). In the GBD, NPC is classified under codes 147–147.9 and 210.7–210.9 in revision 9 of the International Classification of Disease and Injuries (ICD-9), and C11-C11.9 and D10.6 in ICD10^20^. Smoking is defined as the prevalence of current use of any tobacco product and the prevalence of past use of any tobacco product. Among current smokers, it indicates the number of cigarettes smoked per day and the cumulative years of exposure for each smoker, while among former smokers, it estimates the number of years since quitting^21^. ‘smoking-attributable NPC burden’ in this study refers to active smoking exposure.

We extracted mortality rate, YLLs, YLDs, and DALYs for NPC attributable to smoking from 1990 to 2021, using data from 21 GBD super regions and 204 countries and territories. Data were further stratified by year, gender, age, and the sociodemographic index (SDI). The population aged ≥30 years was categorized into 14 age groups: 30–34, 35–39, 40–44, 45–49, 50–54, 55–59, 60–64, 65–69, 70–74, 75–79, 80–84, 85–89, 90–94, and ≥95 years. The SDI is a composite metric of development, calculated as the geometric mean of three factors: fertility rate in individuals under 25 years, education level in those aged ≥15, and per capita income^22^. These factors are normalized on a 0 to 1 scale, where 0 represents minimal health-related development and 1 indicates maximal development. The GBD study categorizes all countries and territories into five SDI levels: low, low-middle, middle, high-middle, and high.

### Statistical analysis

To ensure cross-regional comparability of mortality rates, we calculated age-standardized rates (ASRs) using the World Health Organization standard population. Temporal trends from 1990 to 2021 were quantified using estimated annual percentage changes (EAPC) derived from linear regression models on ln-transformed ASRs. An EAPC >0 indicates an increasing trend, while <0 indicates a decreasing trend. If the 95% CI of the EAPC includes 0, the change is considered statistically insignificant. Based on the EAPC values, we applied hierarchical clustering to classify the death burden change patterns across 21 global regions. The 21 GBD regions were divided into four categories: significant decrease, slight decrease, stable or slight increase, and significant increase. In this study, Euclidean distance matrices were calculated from the research data for cluster analysis, using the Complete Linkage Method as the merging criterion. To identify the optimal number of clusters, the *fviz_nbclust* function from the *factoextra* package was employed to calculate the within-cluster sum of squares (WSS) across different cluster numbers using the Elbow method. To further assess clustering performance, the Silhouette method was used to evaluate the appropriateness of different cluster numbers κ. The silhouette coefficient ranges from -1 to 1, where values closer to 1 indicate better clustering suitability for the data points. The κ with the highest silhouette coefficient was selected as the optimal number of clusters. This approach ensures a robust and valid clustering structure, offering a reliable foundation for subsequent analyses.

Finally, we used the NORDPRED age-period-cohort model to forecast the disease burden from 2022 to 2040. The model was implemented in R using the NORDPRED package, which has shown strong performance in predicting long-term trends in cancer research^23,24^. The model’s core mechanism relies on Poisson regression, integrating age effects (baseline risks across age groups), period effects (influences at specific time points), and cohort effects (risk attributes linked to birth cohorts) to comprehensively analyze historical data and predict future trends.

All visual analyses in this study were performed using R software (version 4.2.3). A p<0.05 was considered statistically significant.

## RESULTS

### Mortality and disease burden of NPC attributable to smoking in 2021

**Figure 1:**
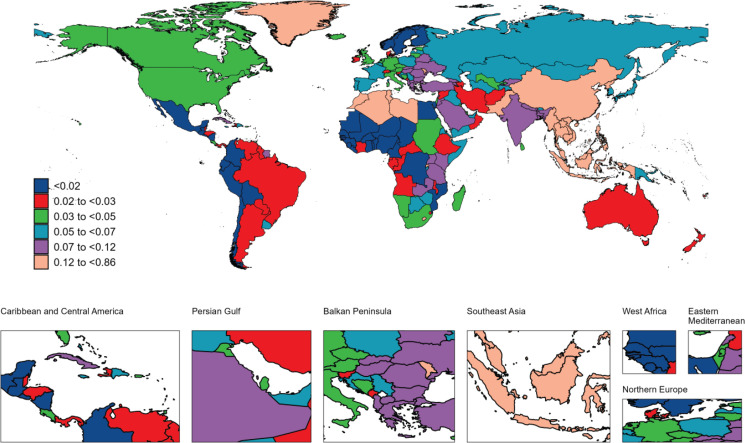
The geographical distribution of age standardized rate of deaths from nasopharyngeal carcinoma attributable to smoking in 2021

**Figure 2:**
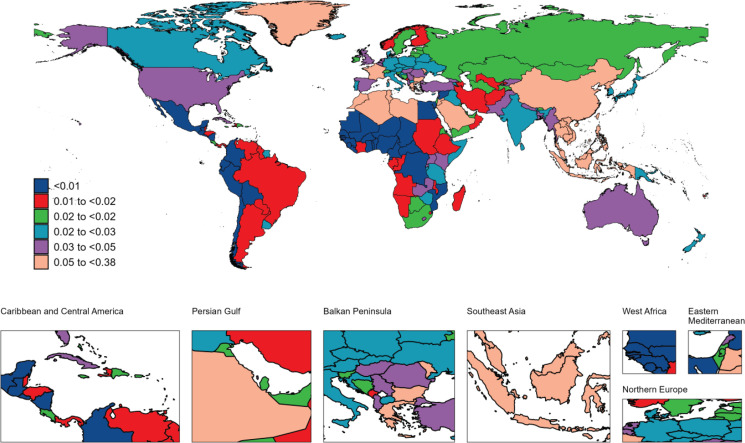
The geographical distribution of age standardized rate of YLDs from nasopharyngeal carcinoma attributable to smoking in 2021

**Figure 3:**
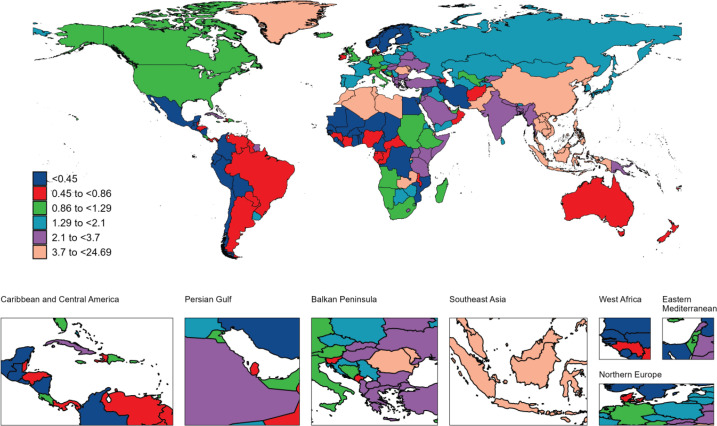
The geographical distribution of age standardized rate of YLLs from nasopharyngeal carcinoma attributable to smoking in 2021

**Figure 4:**
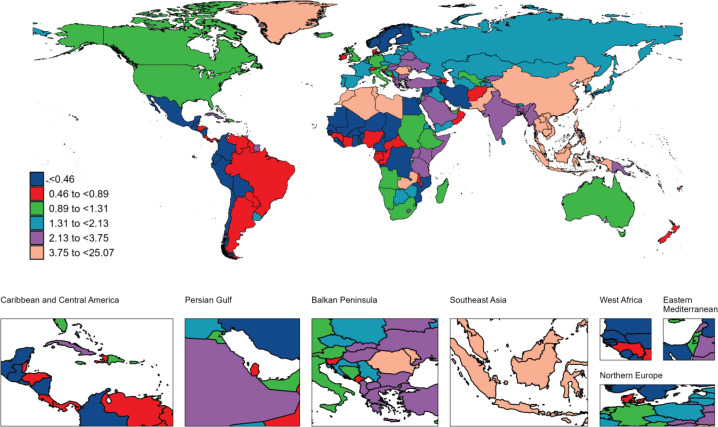
The geographical distribution of age standardized rate of DALYs from nasopharyngeal carcinoma attributable to smoking in 2021

In 2021, there were 13410 smoking-attributed NPC deaths globally, with an ASDR of 0.15 per 100000. Regarding disease burden, the number of YLDs was 10031, with an ASR of 0.11 per 100000; the number of YLLs was 1379583, with an ASR of 4.32 per 100000. Total DALYs amounted to 389614, with an ASR of 4.43 per 100000. Geographically, East Asia had the highest death burden, with 8239 deaths (ASDR=0.37 per 100000), followed by South-East Asia with 1612 deaths (ASDR=0.23 per 100000) and South Asia with 1402 deaths (ASDR=0.09 per 100000). The regional distribution of YLDs, YLLs, and DALYs mirrored that of mortality, with East Asia bearing the highest burden, followed by South-East Asia and South Asia (Tables 1–4). At the national and territories levels, China ranks first in the number of deaths, followed by India. Greenland and Malaysia exhibit the highest age-standardized mortality rates. Additionally, China reports the highest number of YLDs and DALYs. India has the highest number of YLLs, followed by China and Indonesia. Greenland and Malaysia report the highest age-standardized YLL, YLDs and DALYs rates (Figures 1–4).

**Table 1:** The number of deaths and the age-standardized death rate from nasopharyngeal carcinoma attributable to smoking in 1990 and 2021, and trend from 1990 to 2021 globally

*Characteristics*	*1990*		*2021*		*1990–2021*
	*Number of deaths* *(95% UI)*	*Age-standardized* *death rate/100000* *(95% UI)*	*Number of deaths* *(95% UI)*	*Age-standardized* *death rate/100000* *(95% UI)*	*EAPC* *(95% CI)*
**Global**	11942 (15073–8939)	0.29 (0.36–0.22)	13410 (17103–9832)	0.15 (0.20–0.11)	-1.25 (-1.49 – -1.01)
**Sex**					
Female	685 (895–494)	0.03 (0.04–0.02)	560 (761–388)	0.01 (0.02–0.01)	-2.27 (-2.42 – -2.12)
Male	11257 (14239–8419)	0.58 (0.73–0.43)	12850 (16438–9430)	0.31 (0.40–0.23)	-1.18 (-1.42 – -0.94)
**SDI regions**					
High	1547 (1935–1173)	0.15 (0.18–0.11)	1340 (1797–959)	0.07 (0.09–0.05)	-1.42 (-1.49 – -1.34)
High-middle	4230 (5554–3081)	0.41 (0.54–0.30)	4747 (6376–3307)	0.24 (0.32–0.17)	-0.76 (-1.03 – -0.48)
Middle	4853 (6304–3622)	0.44 (0.57–0.33)	5484 (7114–3948)	0.20 (0.26–0.14)	-1.1 (-1.4 – -0.8)
Low-middle	1080 (1416–754)	0.16 (0.22–0.12)	1540 (1987–1098)	0.10 (0.13–0.07)	-0.53 (-0.7 – -0.37)
Low	227 (308–154)	0.09 (0.12–0.06)	292 (402–196)	0.05 (0.07–0.04)	-2.01 (-2.18 – -1.85)
**Region**					
Oceania	4 (6–2)	0.11 (0.16–0.07)	7.04 (10.71–4.28)	0.07 (0.11–0.05)	-0.53 (-0.72 – -0.35)
South-East Asia	842 (1093–611)	0.31 (0.41–0.23)	1612 (2061–1195)	0.23 (0.30–0.17)	0.61 (0.54–0.68)
East Asia	7863 (10263–5737)	0.86 (1.12–0.63)	8239 (11101–5745)	0.37 (0.49–0.26)	-0.91 (-1.25 – -0.57)
Central Asia	27 (35–19)	0.06 (0.07–0.04)	46 (60–33)	0.05 (0.07–0.04)	0.8 (0.57–1.02)
Central Europe	139 (175–106)	0.09 (0.12–0.07)	151 (199–112)	0.08 (0.10–0.06)	0.68 (0.24–1.12)
High-income Asia Pacific	206 (256–157)	0.10 (0.13–0.08)	284 (386–197)	0.06 (0.08–0.04)	0.28 (-0.1–0.67)
Eastern Europe	198 (252–152)	0.07 (0.09–0.05)	211 (275–156)	0.06 (0.08–0.05)	-0.02 (-0.33–0.29)
Australasia	24 (33–17)	0.10 (0.14–0.07)	13 (21–8)	0.03 (0.04–0.02)	-3.33 (-3.4 – -3.25)
Southern Latin America	29 (40–21)	0.06 (0.09–0.04)	19 (27–13)	0.02 (0.03–0.01)	-2.17 (-2.29 – -2.05)
High-income North America	343 (444–250)	0.10 (0.14–0.08)	271 (377–183)	0.04 (0.06–0.03)	-1.71 (-1.82 – -1.61)
Caribbean	17 (23–12)	0.07 (0.09–0.05)	35 (50–24)	0.06 (0.09–0.04)	1.29 (1.07–1.5)
Western Europe	685 (871–512)	0.13 (0.16–0.10)	369 (503–253)	0.04 (0.06–0.03)	-2.48 (-2.54 – -2.43)
Tropical Latin America	41 (53–30)	0.04 (0.05–0.03)	60 (86–40)	0.02 (0.03–0.02)	-0.66 (-1.11 – -0.21)
North Africa and Middle East	290 (383–205)	0.17 (0.22–0.12)	464 (612–327)	0.09 (0.13–0.07)	-0.69 (-0.79 – -0.58)
Central Latin America	22 (28–16)	0.03 (0.03–0.02)	30 (41–20)	0.01 (0.02–0.01)	-0.82 (-1.01 – -0.63)
Andean Latin America	2 (3–1)	0.01 (0.01–0.01)	4 (6–2)	0.01 (0.01–0.00)	0.88 (0.71–1.06)
South Asia	1089 (1456–767)	0.17 (0.23–0.12)	1402 (1835–989)	0.09 (0.12–0.06)	-1 (-1.23 – -0.77)
Eastern Sub-Saharan Africa	75 (101–51)	0.09 (0.12–0.06)	127 (190–76)	0.06 (0.10–0.04)	-1.15 (-1.31 – -0.98)
Central Sub-Saharan Africa	5 (8–3)	0.02 (0.03–0.01)	12 (17–7)	0.02 (0.03–0.01)	-0.38 (-0.72 – -0.04)
Southern Sub-Saharan Africa	23 (30–16)	0.08 (0.11–0.06)	30 (38–21)	0.05 (0.06–0.03)	-0.7 (-0.81 – -0.58)
Western Sub-Saharan Africa	17 (25–12)	0.02 (0.03–0.01)	26 (38–16)	0.01 (0.02–0.01)	-1.94 (-2.06 – -1.81)

UI: uncertainty interval. SDI: sociodemographic index. EAPC: estimated annual percentage change. The 95% CI is used to represent the statistical uncertainty of the EAPC estimate. The 95% UI is used to represent the multidimensional uncertainty of deaths and age-standardized rate, covering factors such as data quality and model error.

**Table 2:** The number of YLDs and the age-standardized YLDs rate from nasopharyngeal carcinoma attributable to smoking in 1990 and 2021, and trend from 1990 to 2021 globally

*Characteristics*	*1990*		*2021*		*1990–2021*
	*Number of YLDs* *(95% UI)*	*Age-standardized* *YLDs rate/100000* *(95% UI)*	*Number of YLDs* *(95% UI)*	*Age-standardized* *YLDs rate/100000* *(95% UI)*	*EAPC* *(95% CI)*
**Global**	5408 (7580–3566)	0.13 (0.18–0.08)	10031 (14649–6576)	0.11 (0.17–0.08)	-0.59 (-0.85 – -0.33)
**Sex**					
Female	333 (475–223)	0.02 (0.02–0.01)	353 (528–227)	0.01 (0.01–0.01)	-2.42 (-2.51 – -2.33)
Male	5075 (7135–3347)	0.25 (0.35–0.16)	9678 (14097–6360)	0.23 (0.33–0.15)	-0.54 (-0.8 – -0.27)
**SDI regions**					
High	960 (1343–659)	0.09 (0.13–0.06)	1022 (1496–666)	0.06 (0.09–0.04)	-1.59 (-1.72 – -1.46)
High-middle	1884 (2737–1207)	0.18 (0.26–0.12)	4438 (6744–2761)	0.24 (0.36–0.15)	0.57 (0.21–0.94)
Middle	2053 (2933–1333)	0.18 (0.25–0.12)	3831 (5610–2476)	0.13 (0.20–0.09)	-1.14 (-1.49 – -0.8)
Low-middle	421 (621–267)	0.06 (0.09–0.04)	621 (907–409)	0.04 (0.06–0.03)	-1.49 (-1.6 – -1.37)
Low	88 (132–55)	0.03 (0.05–0.02)	116 (178–72)	0.02 (0.03–0.01)	-1.93 (-2.04 – -1.83)
**Region**					
Oceania	2 (2–1)	0.05 (0.07–0.03)	3 (5–2)	0.03 (0.05–0.02)	-1.25 (-1.35 – -1.15)
South=East Asia	343 (506–219)	0.12 (0.18–0.08)	756 (1094–494)	0.10 (0.15–0.06)	-0.7 (-0.77 – -0.63)
East Asia	3469 (4888–2220)	0.36 (0.51–0.23)	7473 (11152–4737)	0.34 (0.51–0.22)	-0.43 (-0.87–0.01)
Central Asia	11 (15–7)	0.02 (0.03–0.01)	18 (28–12)	0.02 (0.03–0.01)	0.06 (-0.11–0.23)
Central Europe	58 (81–39)	0.04 (0.05–0.03)	70 (101–46)	0.04 (0.05–0.02)	-0.09 (-0.54–0.36)
High-income Asia Pacific	91 (129–62)	0.04 (0.06–0.03)	123 (179–81)	0.03 (0.05–0.02)	-1.36 (-1.79 – -0.93)
Eastern Europe	78 (113–52)	0.03 (0.04–0.02)	84 (121–55)	0.03 (0.04–0.02)	-0.74 (-1.06 – -0.42)
Australasia	27 (41–18)	0.12 (0.18–0.08)	20 (32–12)	0.05 (0.07–0.03)	-3.25 (-3.43 – -3.07)
Southern Latin America	12 (18–8)	0.03 (0.04–0.02)	9 (14–5)	0.01 (0.02–0.01)	-2.71 (-2.82 – -2.61)
High-income North America	306 (442–205)	0.10 (0.14–0.06)	288 (433–187)	0.05 (0.07–0.03)	-2.33 (-2.45 – -2.21)
Caribbean	7 (10–4)	0.03 (0.04–0.02)	14 (23–9)	0.03 (0.04–0.02)	0.04 (-0.21–0.28)
Western Europe	394 (551–268)	0.08 (0.11–0.05)	283 (426–182)	0.04 (0.06–0.03)	-2.2 (-2.38 – -2.02)
Tropical Latin America	17 (24–11)	0.02 (0.02–0.01)	25 (39–16)	0.01 (0.01–0.01)	-2.42 (-2.91 – -1.94)
North Africa and Middle East	116 (169–76)	0.06 (0.09–0.04)	215 (321–139)	0.04 (0.06–0.03)	-1.47 (-1.54 – -1.4)
Central Latin America	8 (12–6)	0.01 (0.01–0.01)	12 (18–8)	0.00 (0.01–0.00)	-2.8 (-3.02 – -2.58)
Andean Latin America	1 (1–0)	0.00 (0.01–0.00)	2 (3–1)	0.00 (0.00–0.00)	-0.7 (-0.85 – -0.55)
South Asia	423 (625–271)	0.07 (0.10–0.05)	558 (829–364)	0.04 (0.05–0.02)	-2.12 (-2.29 – -1.95)
Eastern Sub-Saharan Africa	29 (45–19)	0.03 (0.05–0.02)	51 (85–29)	0.02 (0.04–0.01)	-1.24 (-1.32 – -1.16)
Central Sub-Saharan Africa	2 (3–1)	0.01 (0.01–0.00)	5 (8–3)	0.01 (0.01–0.00)	-0.4 (-0.64 – -0.15)
Southern Sub-Saharan Africa	9 (13–6)	0.03 (0.05–0.02)	12 (18–8)	0.02 (0.03–0.01)	-1.75 (-1.88 – -1.62)
Western Sub-Saharan Africa	7 (10–4)	0.01 (0.01–0.00)	11 (18–6)	0.00 (0.01–0.00)	-1.77 (-1.91 – -1.62)

UI: uncertainty intervals. SDI: sociodemographic index. EAPC: estimated annual percentage change. The 95% CI is used to represent the statistical uncertainty of the EAPC estimate. Τhe 95% UI is used to represent the multidimensional uncertainty of YLDs and age-standardized rate, covering factors such as data quality and model error.

**Table 3:** The number of YLLs and the age-standardized YLLs rate from nasopharyngeal carcinoma attributable to smoking in 1990 and 2021, and trend from 1990 to 2021 globally

*Characteristics*	*1990*		*2021*		*1990–2021*
	*Number of YLLs* *(95% UI)*	*Age-standardized* *YLLs rate/100000* *(95% UI)*	*Number of YLLs* *(95% UI)*	*Age-standardized* *YLLs rate/100000* *(95% UI)*	*EAPC* *(95% CI)*
**Global**	370411 (465971–277842)	8.66 (10.89–6.50)	1379583 (484268–279023)	4.32 (5.51–3.17)	-2.61 (-2.81 – -2.42)
**Sex**					
Female	18965 (24799–13867)	0.88 (1.14–0.64)	14397 (19308–10093)	0.32 (0.42–0.22)	-3.59 (-3.72 – -3.46)
Male	351445 (444282–263766)	16.95 (21.42–12.70)	365186 (467205–268335)	8.58 (10.97–6.31)	-2.56 (-2.77 – -2.36)
**SDI regions**					
High	45358 (56455–34361)	4.38 (5.45–3.32)	33887 (44395–24357)	1.90 (2.48–1.37)	-2.93 (-3.01 – -2.84)
High-middle	130479 (170666–94349)	12.53 (16.38–9.07)	134020 (180580–93371)	6.92 (9.34–4.82)	-2.42 (-2.66 – -2.18)
Middle	152645 (198349–112801)	12.80 (16.66–9.50)	155640 (202076–112395)	5.42 (7.02–3.92)	-3.15 (-3.4 – -2.89)
Low-middle	34421 (45221–24265)	4.86 (6.38–3.41)	46560 (60106–33249)	2.92 (3.77–2.09)	-1.7 (-1.81 – -1.6)
Low	7355 (10013–4978)	2.75 (3.74–1.87)	9279 (12866–6164)	1.54 (2.12–1.03)	-2.11 (-2.22 – -2.01)
**Region**					
Oceania	131 (186–79)	3.53 (5.02–2.15)	244 (371–147)	2.46 (3.76–1.50)	-1.26 (-1.37 – -1.14)
South-East Asia	26423 (34300–19051)	8.99 (11.67–6.52)	49444 (63486–36793)	6.68 (8.58–4.97)	-1.15 (-1.22 – -1.07)
East Asia	245462 (319843–178264)	24.87 (32.44–18.15)	229053 (311677–160498)	10.22 (13.90–7.19)	-3.38 (-3.7 – -3.05)
Central Asia	855 (1102–628)	1.68 (2.16–1.23)	1399 (1851–1024)	1.50 (1.98–1.10)	-0.2 (-0.36 – -0.04)
Central Europe	4323 (5416–3303)	2.88 (3.61–2.21)	4289 (5530–3196)	2.29 (2.94–1.71)	-0.72 (-1.16 – -0.27)
High-income Asia Pacific	5814 (7210–4449)	2.78 (3.45–2.13)	5734 (7555–4023)	1.47 (1.90–1.05)	-2.52 (-2.89 – -2.15)
Eastern Europe	6333 (8111–4867)	2.27 (2.91–1.74)	6371 (8245–4715)	1.98 (2.56–1.46)	-0.95 (-1.28 – -0.61)
Australasia	663 (891–476)	2.92 (3.92–2.10)	357 (541–223)	0.80 (1.20–0.51)	-4.24 (-4.32 – -4.16)
Southern Latin America	883 (1168–629)	1.88 (2.49–1.34)	524 (734–360)	0.63 (0.89–0.44)	-3.28 (-3.38 – -3.17)
High-income North America	9764 (12561–7220)	3.12 (3.99–2.30)	7044 (9550–4887)	1.19 (1.60–0.83)	-3.26 (-3.32 – -3.19)
Caribbean	449 (597–324)	1.71 (2.28–1.23)	879 (1247–621)	1.62 (2.30–1.14)	-0.24 (-0.48–0.01)
Western Europe	19257 (24331–14576)	3.77 (4.75–2.87)	9170 (12217–6428)	1.22 (1.62–0.87)	-3.67 (-3.74 – -3.61)
Tropical Latin America	1331 (1707–1003)	1.27 (1.64–0.95)	1739 (2425–1172)	0.65 (0.91–0.44)	-2.84 (-3.34 – -2.34)
North Africa and Middle East	8911 (11662–6341)	4.63 (6.09–3.28)	13776 (18049–9853)	2.64 (3.47–1.87)	-2.03 (-2.12 – -1.94)
Central Latin America	609 (789–444)	0.68 (0.88–0.49)	786 (1068–549)	0.30 (0.41–0.21)	-3.07 (-3.29 – -2.85)
Andean Latin America	53 (73–37)	0.25 (0.34–0.17)	102 (149–67)	0.17 (0.25–0.11)	-1.06 (-1.22 – -0.91)
South Asia	35199 (46724–24868)	5.15 (6.86–3.63)	42041 (55067–29570)	2.59 (3.39–1.82)	-2.34 (-2.5 – -2.18)
Eastern Sub-Saharan Africa	2478 (3336–1694)	2.75 (3.71–1.88)	4323 (6553–2558)	1.99 (2.99–1.19)	-1.28 (-1.36 – -1.2)
Central Sub-Saharan Africa	179 (257–113)	0.65 (0.92–0.41)	395 (598–237)	0.54 (0.81–0.33)	-0.44 (-0.67 – -0.21)
Southern SubSaharan Africa	694 (913–488)	2.31 (3.08–1.61)	945 (1213–674)	1.43 (1.83–1.02)	-1.74 (-1.87 – -1.61)
Western Sub-Saharan Africa	599 (851–404)	0.57 (0.82–0.39)	968 (1386–579)	0.36 (0.52–0.22)	-1.79 (-1.94 – -1.63)

UI: uncertainty intervals. SDI: sociodemographic index. EAPC: estimated annual percentage change. The 95% CI is used to represent the statistical uncertainty of the EAPC estimate. Τhe 95% UI is used to represent the multidimensional uncertainty of YLLs and age-standardized rate, covering factors such as data quality and model error.

**Table 4:** The number of DALYs and the age-standardized DALYs rate from nasopharyngeal carcinoma attributable to smoking in 1990 and 2021, and trend from 1990 to 2021 globally

*Characteristics*	*1990*		*2021*		*1990–2021*
	*Number of DALYs* *(95% UI)*	*Age-standardized* *DALYs* *rate/100000* *(95% UI)*	*Number of DALYs* *(95% UI)*	*Age-standardized* *DALYs* *rate/100000 (95% UI)*	*EAPC* *(95% CI)*
**Global**	375818 (472216– 282167)	8.79 (11.04–6.59)	389614 (495582–286016)	4.43 (5.64–3.25)	-2.58 (-2.77 – -2.38)
**Sex**					
Female	19298 (25214–14151)	0.89 (1.16–0.65)	14750 (19734–10359)	0.32 (0.43–0.23)	-3.57 (-3.69 – -3.44)
Male	356521 (450717–267635)	17.20 (21.73–12.89)	374864 (477057–275073)	8.81 (11.22–6.47)	-2.52 (-2.73 – -2.32)
**SDI regions**					
High	46318 (57530–35186)	4.47 (5.57–3.40)	34909 (45768–25071)	1.96 (2.55–1.41)	-2.89 (-2.98 – -2.81)
High-middle	132363 (172877–95647)	12.71 (16.59–9.19)	138458 (186729–96546)	7.15 (9.66–4.99)	-2.36 (-2.6 – -2.11)
Middle	154698 (200954–114510)	12.98 (16.89–9.63)	159471 (206908–115216)	5.56 (7.19–4.02)	-3.11 (-3.37 – -2.85)
Low-middle	34841 (45749–24564)	4.93 (6.46–3.46)	47181 (60909–33668)	2.96 (3.83–2.12)	-1.7 (-1.8 – -1.59)
Low	7443 (10126–5038)	2.78 (3.79–1.89)	9395 (13037–6236)	1.56 (2.16–1.05)	-2.11 (-2.21 – -2.01)
**Region**					
Oceania	133 (188–80)	3.57 (5.09–2.18)	247 (375–149)	2.50 (3.80–1.52)	-1.26 (-1.37 – -1.14)
South-East Asia	26766 (34788–19293)	9.11 (11.83–6.60)	50200 (64542–37415)	6.79 (8.73–5.06)	-1.14 (-1.21 – -1.07)
East Asia	248931 (324196–181147)	25.23 (32.90–18.46)	236526 (320334–166175)	10.57 (14.30–7.43)	-3.31 (-3.64 – -2.99)
Central Asia	866 (1118–635)	1.70 (2.20–1.24)	1418 (1878–1038)	1.52 (2.01–1.11)	-0.2 (-0.36 – -0.04)
Central Europe	4380 (5479–3342)	2.92 (3.65–2.23)	4359 (5619–3250)	2.33 (2.99–1.74)	-0.71 (-1.15 – -0.26)
High-income Asia Pacific	10070 (12964– 7481)	2.82 (3.50–2.17)	7332 (9975–5091)	1.50 (1.94–1.07)	-2.5 (-2.87 – -2.13)
Eastern Europe	6411 (8226–4924)	2.29 (2.95–1.76)	6454 (8355–4791)	2.00 (2.60–1.49)	-0.94 (-1.28 – -0.61)
Australasia	690 (927–495)	3.04 (4.08–2.18)	376 (571–236)	0.84 (1.27–0.54)	-4.2 (-4.28 – -4.12)
Southern Latin America	895 (1183–637)	1.91 (2.53–1.36)	533 (747–367)	0.64 (0.90–0.44)	-3.27 (-3.37 – -3.17)
High-income North America	5905 (7320–4531)	3.21 (4.11–2.38)	5857 (7734–4108)	1.24 (1.67–0.87)	-3.22 (-3.28 – -3.16)
Caribbean	456 (607–329)	1.73 (2.31–1.25)	894 (1272–630)	1.65 (2.35–1.16)	-0.23 (-0.48–0.01)
Western Europe	19651 (24829–14933)	3.85 (4.84–2.94)	9453 (12581–6631)	1.26 (1.67–0.90)	-3.64 (-3.71 – -3.57)
Tropical Latin America	1348 (1728–1014)	1.29 (1.66–0.96)	1764 (2463–1189)	0.66 (0.93–0.45)	-2.83 (-3.33 – -2.33)
North Africa and Middle East	9027 (11810–6429)	4.69 (6.17–3.33)	13992 (18345–10009)	2.68 (3.52–1.90)	-2.02 (-2.11 – -1.93)
Central Latin America	617 (799–451)	0.68 (0.89–0.50)	798 (1085–557)	0.31 (0.42–0.21)	-3.07 (-3.28 – -2.85)
Andean Latin America	54 (74–38)	0.25 (0.34–0.17)	104 (151–68)	0.17 (0.25–0.11)	-1.06 (-1.22 – -0.9)
South Asia	35621 (47244–25184)	5.22 (6.95–3.68)	42598 (55819–30036)	2.62 (3.43–1.85)	-2.34 (-2.5 – -2.18)
Eastern Sub-Saharan Africa	2507 (3367–1713)	2.79 (3.74–1.90)	4374 (6631– 2590)	2.02 (3.03–1.21)	-1.28 (-1.36 – -1.2)
Central Sub-Saharan Africa	181 (259–114)	0.66 (0.93–0.41)	400 (606–240)	0.55 (0.82–0.34)	-0.44 (-0.67 – -0.21)
Southern Sub-Saharan Africa	703 (926–494)	2.34 (3.12–1.63)	957 (12301–684)	1.44 (1.86–1.03)	-1.74 (-1.87 – -1.61)
Western Sub-Saharan Africa	605 (860–409)	0.58 (0.82–0.39)	979 (1403–587)	0.36 (0.53–0.22)	-1.79 (-1.94 – -1.63)

UI: uncertainty intervals. SDI: sociodemographic index. EAPC: estimated annual percentage change. The 95% CI is used to represent the statistical uncertainty of the EAPC estimate. Τhe 95% UI is used to represent the multidimensional uncertainty of DALYs and age-standardized rate, covering factors such as data quality and model error.

In 2021, age and gender analyses highlighted substantial disparities. Globally, male deaths totaled 12850 (ASDR=0.31 per 100000), far exceeding the 560 deaths among women (ASDR=0.01 per 100000). Trends in YLDs, YLLs, and DALYs also showed a significantly higher burden in men compared to women (Tables 1–4). Across age groups, male mortality rose gradually from the age group of 30–34 years, peaking at the age of 65–69 years and then gradually declining. In contrast, female mortality showed a relatively stable distribution, remaining significantly lower than male mortality. YLDs peaked in younger age groups, with men reaching their highest level in the age group of 50–54 years, while women remained relatively stable at a lower level. YLLs exhibited a marked gender disparity, with men peaking in the age group of 55–59 years and forming a distinct plateau between the ages of 45 and 75 years. DALYs peaked in the age group of 55–59 years, reflecting significant gender differences, with men forming a broad peak period, while women remained at lower levels (Supplementary file Figures S1–S4). A further comparison with 1990 data indicated that this age-gender distribution pattern remained stable, with significant gender differences maintained across all indicators (Supplementary file Figures S5–S8).

From a socioeconomic perspective, medium SDI regions recorded the highest number of deaths, followed by high-middle SDI regions. Low SDI regions reported the fewest cases. For YLDs, high-middle SDI and medium SDI regions ranked first and second, with 4438 cases and 3831 cases, respectively, while low SDI regions had the lowest burden. Regarding YLLs, medium SDI regions contributed the highest burden, followed by high-middle SDI regions. For DALYs, medium SDI and high-middle SDI regions bore a heavier burden, whereas high SDI and low SDI regions had relatively lighter burdens (Tables 1–4).

Supplementary file Figures S9–S12 illustrate the nonlinear relationships between ASRs of mortality, YLDs, YLLs, and DALYs with SDI. These indicators exhibit an inverted ‘U’-shaped curve with SDI. East Asia showed the highest age-standardized rates, which significantly decreased with rising SDI values. Other regions exhibited different patterns: low SDI regions (SDI=0.3–0.5) maintained relatively low levels with a gradual increasing trend, peaking at an SDI of 0.5. Medium SDI regions (SDI=0.5–0.7) showed a slight increase or stabilization, while high SDI regions (SDI >0.7) generally exhibited a declining trend.

### Temporal trend for mortality and disease burden of NPC attributable to smoking from 1990 to 2021

Globally, smoking attributed NPC deaths increased from 11942 in 1990 to 13410 in 2021. However, the ASDR decreased from 0.29 per 100000 to 0.15 per 100000, with an EAPC of -1.25. Similarly, the absolute number of YLDs increased from 5408 in 1990 to 10031 in 2021, reflecting an increase number of affected individuals, while the age-standardized YLD rate decreased from 0.13 per 100000 to 0.11 per 100000, with an EAPC of -0.59. YLLs increased from 370411 in 1990 to 1379583 in 2021, while the age-standardized YLL rate decreased from 8.66 per 100000 to 4.32 per 100000 (EAPC= -2.61). DALYs increased from 375818 in 1990 to 389614 in 2021, whereas the ASR decreased from 8.79 per 100000 to 4.43 per 100000 (EAPC= -2.58). These overall trends indicate that although the absolute global burden of smoking attributed NPC has increased, the age-standardized burden has declined (Tables 1–4; and Supplementary file Figure S13).

Gender-stratified analysis revealed that males exhibited significantly higher burden indicators across all metrics compared to females from 1990 to 2021. However, the reduction in burden was more pronounced in females (Tables 1–4; and Supplementary file Figures S1–S8, S14). From 1990 to 2021, significant declines in mortality, YLLs, and DALYs were observed globally across different SDI regions. The most significant reduction in mortality occurred in low-SDI regions (EAPC= -2.01), followed by high-SDI regions (EAPC= -1.42). YLLs and DALYs saw the most pronounced decreases in middle-SDI and high-SDI regions. YLDs generally declined across SDI regions, except for a slight increase in middle-high SDI regions (Tables 1–4; and Supplementary file Figure S15). The changes in the burden of smoking attributed NPC show significant regional differences. Australasia and Western Europe exhibited the most substantial declines in deaths, YLLs and DALYs, while the Caribbean and Central Asia showed increasing trends. For YLDs, Australasia and Southern Latin America demonstrated the best performance (Tables 1–4).

A cluster analysis was performed based on the EAPC of death rates, grouping regions with similar patterns. As shown in Supplementary file Figure S16A, the 21 regions were categorized into four groups. The first group, which includes regions such as Australasia, East Asia and Western Europe, is characterized by relatively low baseline levels and a rapid decline in mortality. The second group, shown in yellow in the figure, represents countries with a downward trend. The third group exhibits a slight increase or stable trend. The fourth group, which includes the Caribbean and Central Asia, has relatively low baseline levels but a clear upward trend in mortality.

### The predicted results from 2022 to 2044

Based on historical data from 1990 to 2021, the NORDPRED age-period-cohort model was used to project the burden of smoking-related NPC from 2022 to 2040. The results indicate that the number of deaths, YLDs, YLLs, and DALYs will increase. However, age-standardized rates of mortality, YLLs, and DALYs are expected to decline, while age-standardized YLDs may increase (Supplementary file Figure S16B).

## DISCUSSION

Tobacco is classified as a Group 1 carcinogen by the International Agency for Research on Cancer (IARC)^25^. Globally, in 2020, an estimated 1.18 billion people regularly used some form of tobacco, resulting in 7.0 million deaths, accounting for approximately one-seventh of all deaths that year^16^. Smoking has been identified as a major risk factor for the development, progression, and prognosis of NPC. Studies have shown that smoking promotes the development of NPC by damaging DNA and causing genetic mutations^26,27^, inducing chronic inflammation that fosters tumor formation^28^, and synergizing with Epstein-Barr virus (EBV) to drive carcinogenesis^29^. However, there have been no previous reports on the mortality and disease burden of smoking attributed NPC, highlighting a lack of sufficient research to guide the development of precise policies. This study systematically analyzed the global burden of smoking attributed NPC deaths and their trends from 1990 to 2021 using GBD data and projected the disease burden from 2022 to 2040. By examining key indicators such as mortality, YLDs, YLLs, DALYs, and EAPC, it provides the first comprehensive evaluation of the impact of smoking on the burden of NPC deaths. Furthermore, clustering similar regions enables accurate identification of key areas for targeted prevention and control, thereby guiding the development of region-specific control policies.

Our findings revealed 13410 smoking attributed NPC deaths worldwide in 2021, with an ASMR of 0.15 per 100000. It has been reported that in 2022, there were approximately 73476 deaths from NPC^1^. It is estimated that smoking attributed deaths accounted for about 18% of the total. This highlights that smoking, as an environmental factor, contributes significantly to the mortality burden of NPC and warrants greater attention. Geographically, the highest burden was observed in East Asia, followed by South-East Asia and South Asia. These geographical patterns closely align with regional smoking prevalence trends and the characteristics of NPC incidence^30,31^. Gender disparity analysis revealed significant imbalance, with the ASDR for males being approximately 31 times higher than that for females. However, the reduction in burden was more pronounced among women, likely due to the reduction in smoking rates, the effectiveness of public health interventions, and females’ greater propensity to quit smoking. With advancements in early screening and treatment technologies, females have also experienced significant improvements in the smoking-related NPC burden. This pronounced difference reflects the traditionally higher prevalence of smoking among males and underscores the necessity of targeted interventions focusing on male populations.

From 1990 to 2021, the global ASDR for smoking attributed NPC showed a significant decline. However, substantial regional disparities were observed. Australasia achieved the greatest progress, likely due to its comprehensive tobacco control policies and robust healthcare systems, while the Caribbean showed an upward trend, indicating potential challenges in tobacco control in this region. In terms of socioeconomic development levels, middle and high-middle SDI regions reported the highest number of deaths, accounting for 76% of all cases. In contrast, high and low SDI regions showed the fastest declines in mortality (EAPC= -2.01 and -1.42, respectively). This pattern does not fully align with the epidemiological statistics of smoking prevalence, as WHO reports that less developed regions have the highest smoking populations^15^. This suggests that the distribution of smoking attributed NPC deaths may be influenced by the geographical distribution of its incidence, with high-incidence areas such as East Asia playing a significant role. Moreover, economic development alone is not the sole determinant of the rate of disease burden improvement; it is the result of multiple interacting factors.

In addition to controlling mortality, a comprehensive assessment of disease burden is equally important. Studies have shown that smoking reduces the sensitivity to radiotherapy and chemotherapy, increases the risk of treatment-related complications, and significantly affects patients’ quality of life and long-term survival^13,^ ^32-34^. These factors substantially contribute to the overall disease burden in the population. In 2021, the global YLDs rate was 0.11 per 100000, the YLLs rate was 4.5 per 100000, and the DALYs rate was 4.5 per 100000, reflecting a substantial disease burden. From 1990 to 2021, these indicators showed a declining trend. Although our data indicate that global NPC prevention and smoking cessation measures have achieved positive outcomes in improving patient quality of life and extending survival, long-term prevention and control efforts are still needed to further reduce the disease burden.

Given the uneven geographical distribution of NPC and the regional clustering of smoking prevalence, cluster analysis may help group countries with similar epidemiological profiles. This approach could facilitate the development and implementation of more targeted prevention and control strategies. The successful experiences of the first group of regions (e.g. Australasia, Western Europe) are worth emulating, as their low baseline levels and rapid declines are likely associated with well-established public health systems and effective tobacco control measures. In contrast, the upward trends in the fourth group (e.g. the Caribbean, Central Asia) highlight the need for increased attention and support in these regions.

According to the projections, the disease burden is expected to exhibit new characteristics from 2022 to 2040. While age-standardized rates may continue to decline, the absolute number of deaths, YLLs and DALYs are projected to increase. This seemingly paradoxical trend primarily reflects the impact of population aging, suggesting a sustained increase in healthcare demand. Notably, both the number and the age-standardized rate of YLDs are projected to increase. This trend highlights the need to strengthen chronic disease management and rehabilitation measures, placing greater demands on healthcare systems and resource allocation. Furthermore, the projection emphasizes the importance of preventive measures and the urgent need to enhance patients’ quality of life and mitigate long-term health losses.

### Strengths and limitations

The strength of this study lies in its inclusion of comprehensive global data on smoking-related NPC from 1990 to 2021, encompassing a 31-year longterm trend analysis. As the first study specifically addressing smoking-attributable NPC mortality and disease burden using GBD data, our findings provide valuable epidemiological insights. However, several limitations warrant consideration. First, the quality of GBD data may vary across regions, particularly in areas with limited healthcare resources, potentially leading to underreporting. Second, our EAPC trend analysis relies on linear regression assumptions that may not capture complex temporal patterns. Third, the smoking-attributable fractions may be influenced by several unmeasured factors, such as: 1) lack of granular data on smoking intensity, duration, and cessation history; 2) exclusion of secondhand smoke and emerging e-cigarette exposures; and 3) potential interactions with other NPC risk factors (e.g. EBV infection patterns, occupational carcinogens). Our clustering analysis, while informative, has inherent limitations in centroid selection sensitivity that may affect regional pattern interpretations. Fourth, although we adjusted for major confounders through GBD’s standard methodology, residual confounding may persist from unaccounted environmental factors (e.g. indoor air pollution from cooking fuels, formaldehyde exposure) that show gender/regional variations in exposure patterns. Lastly, significant changes in future tobacco control policies could result in actual trends deviating from the projections, as the predictive model used in this study does not fully account for such potential policy shifts. Therefore, these findings should be interpreted with caution and regularly updated as new data become available.

## CONCLUSIONS

This study provides critical evidence for understanding the evolution of the global burden of smoking attributed NPC. The observed significant regional and gender disparities, along with the projected increase in disease burden, present new challenges for global tobacco control and NPC prevention. This study underscores the importance of strengthening tobacco control measures, particularly those targeting high-burden regions and high-risk populations. Additionally, it highlights the need for enhanced regional experience-sharing and policy coordination to achieve more effective disease prevention and control.

## Supplementary Material



## Data Availability

The data supporting this research are available from the following source: https://vizhub.healthdata.org/gbd-results/.
