# Supplementary material for: Global, regional, and national temporal trends in mortality and disease burden of nasopharyngeal carcinoma attributable to smoking from 1990 to 2021 and predictions to 2040
Source: Tob Induc Dis. 2025 Jun 30;23:10.18332/tid/204742. doi: 10.18332/tid/204742 (PMC12207875; doi:10.18332/tid/204742)
Supplement: Supplementary file 1 [file TID-23-86-s1.pdf]

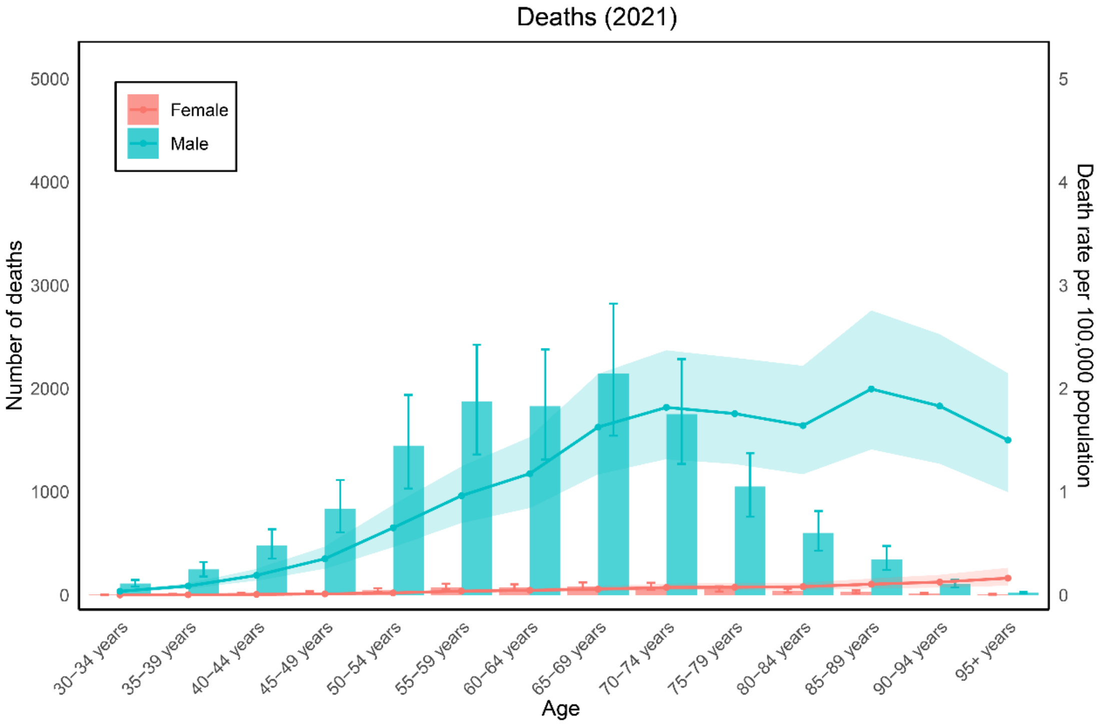

Figure S1. Number and age standardized rate of deaths of NPC attributable to smoking by gender and age in 2021.

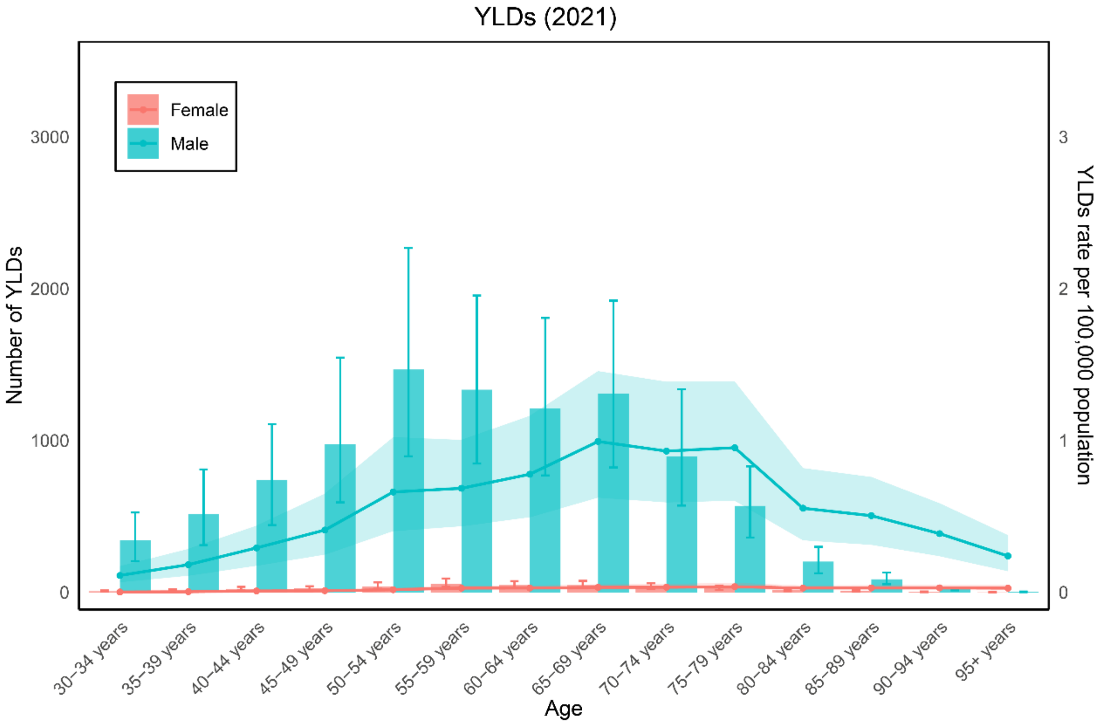

Figure S2. Number and age standardized rate of YLDs of NPC attributable to smoking by gender and age in 2021.

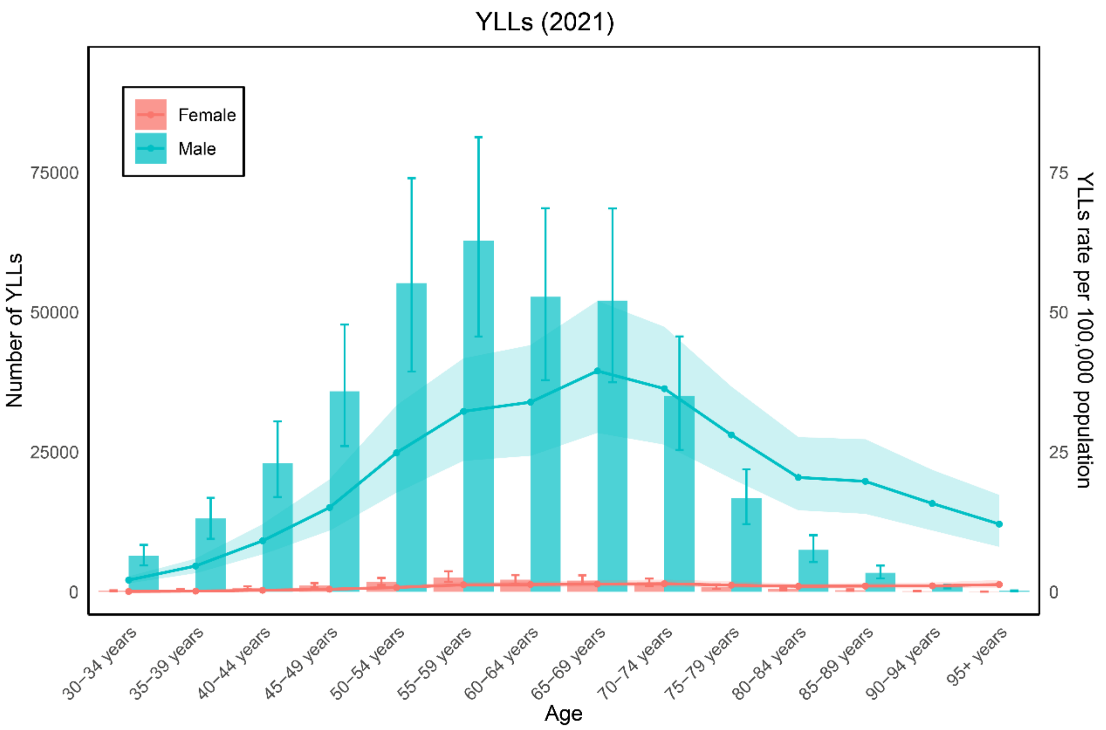

Figure S3. Number and age standardized rate of YLLs of NPC attributable to smoking by gender and age in 2021.

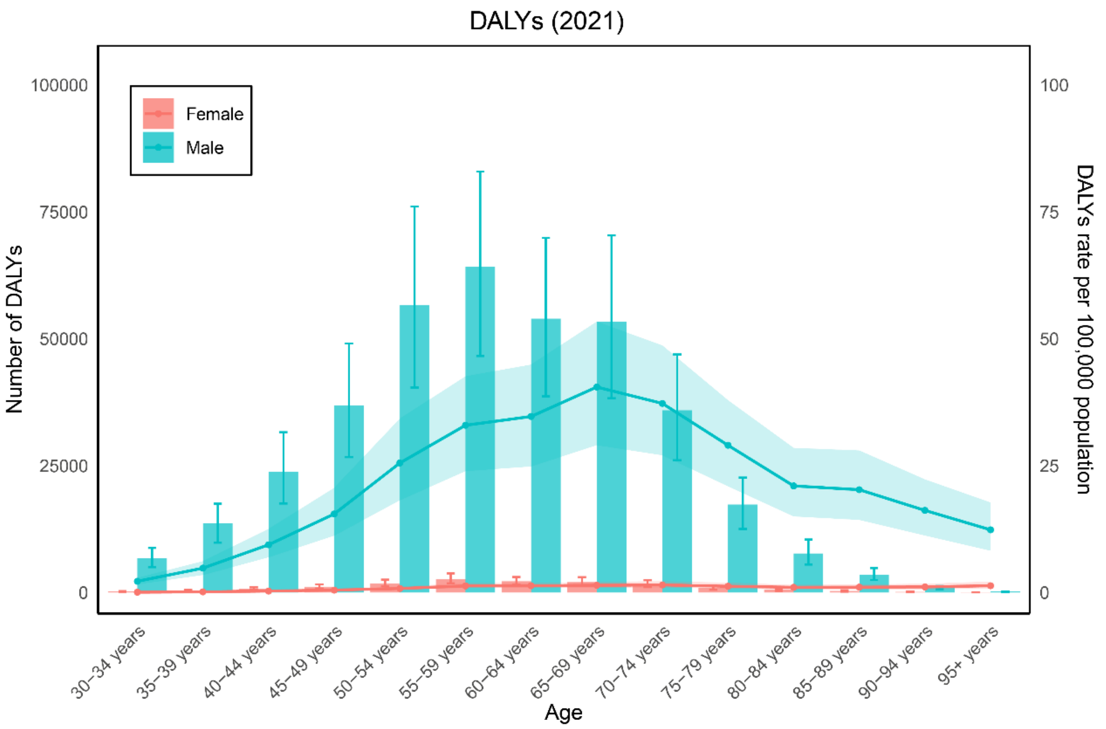

Figure S4. Number and age standardized rate of DALYs of NPC attributable to smoking by gender and age in 2021.

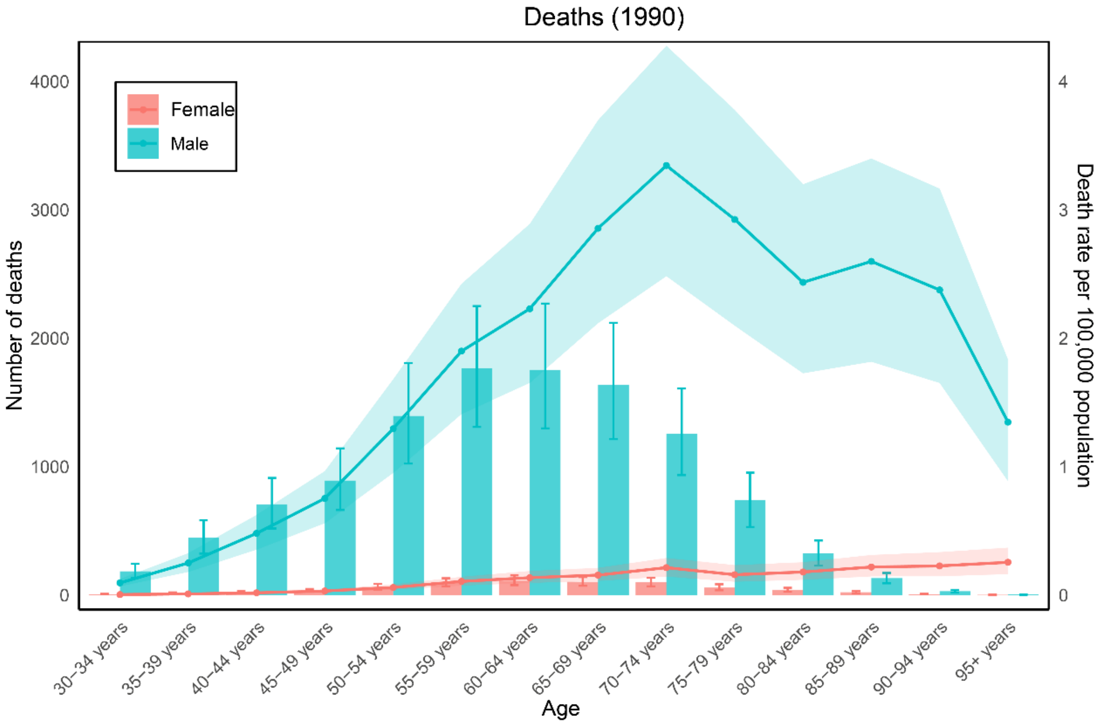

Figure S5. Number and age standardized rate of deaths of NPC attributable to smoking by gender and age in 1990.

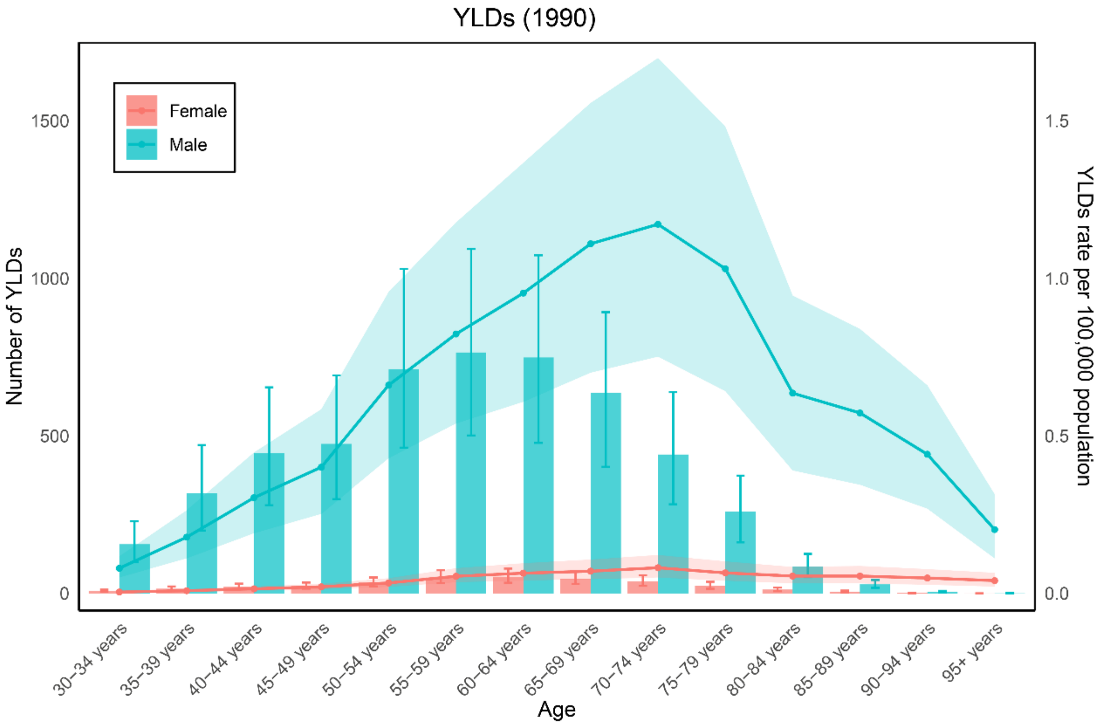

Figure S6. Number and age standardized rate of YLDs of NPC attributable to smoking by gender and age in 1990.

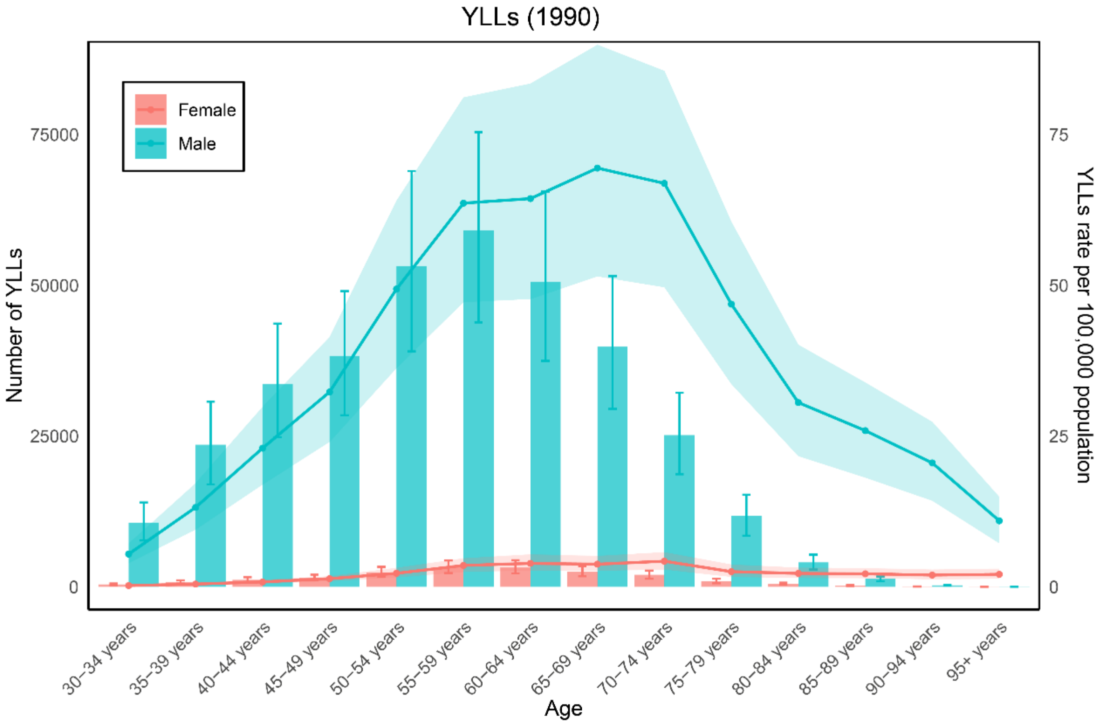

Figure S7. Number and age standardized rate of YLLs of NPC attributable to smoking by gender and age in 1990.

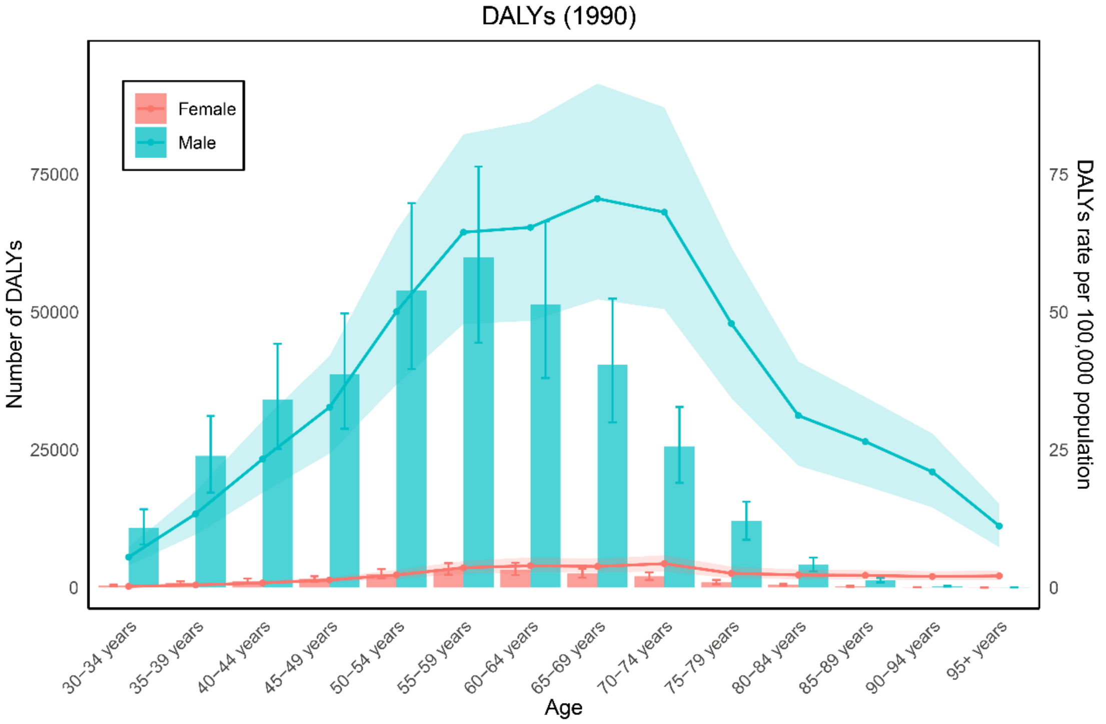

Figure S8. Number and age standardized rate of DALYs of NPC attributable to smoking by gender and age in 1990.

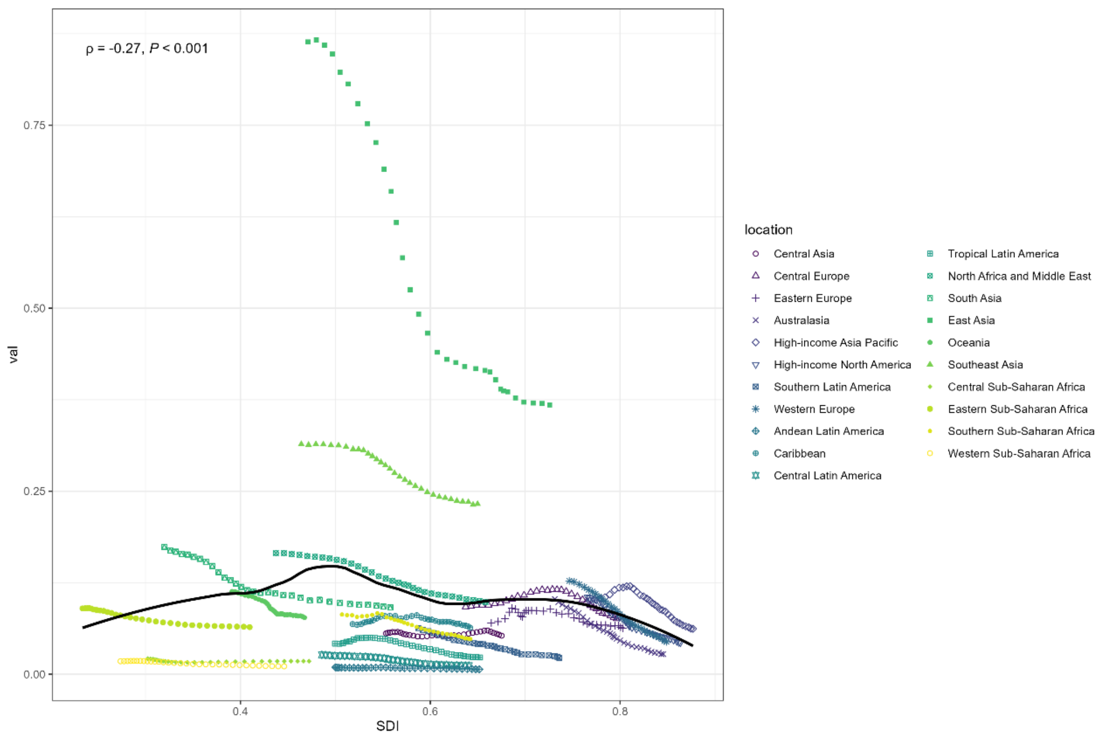

Figure S9. Age-standardized death rates of NPC attributable to smoking across 21 GBD regions by socio-demographic index from 1990 to 2021.

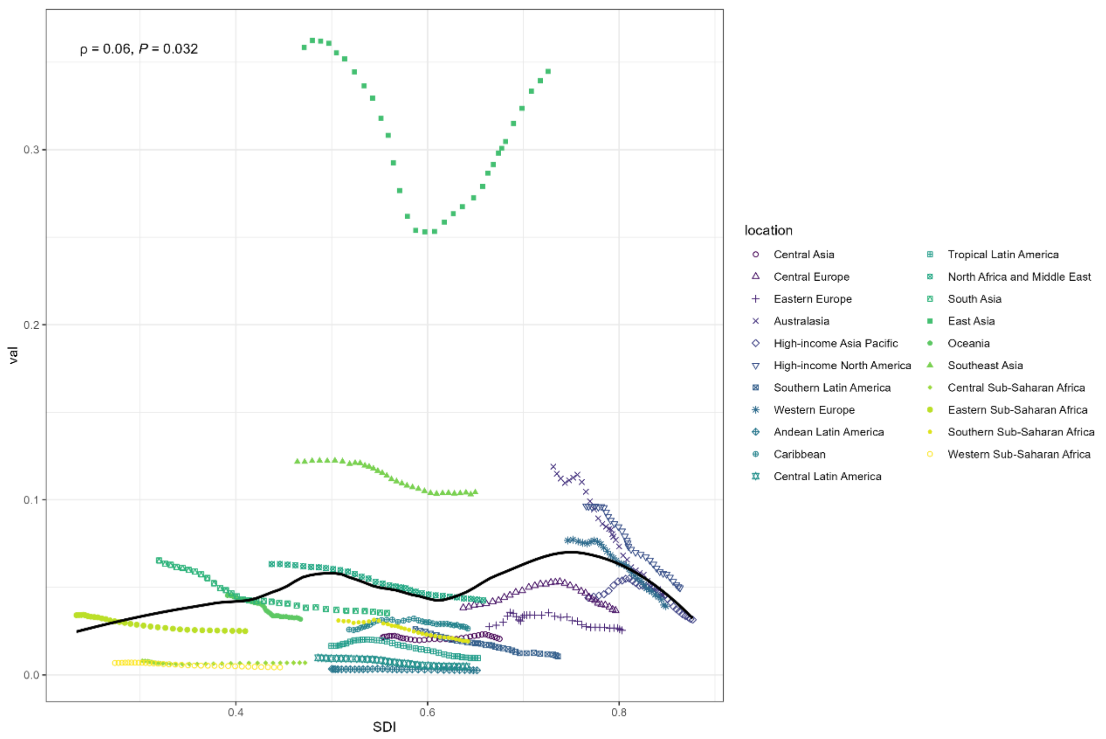

Figure S10. Age-standardized rates of YLDs of NPC attributable to smoking across 21 GBD regions by socio-demographic index from 1990 to 2021.

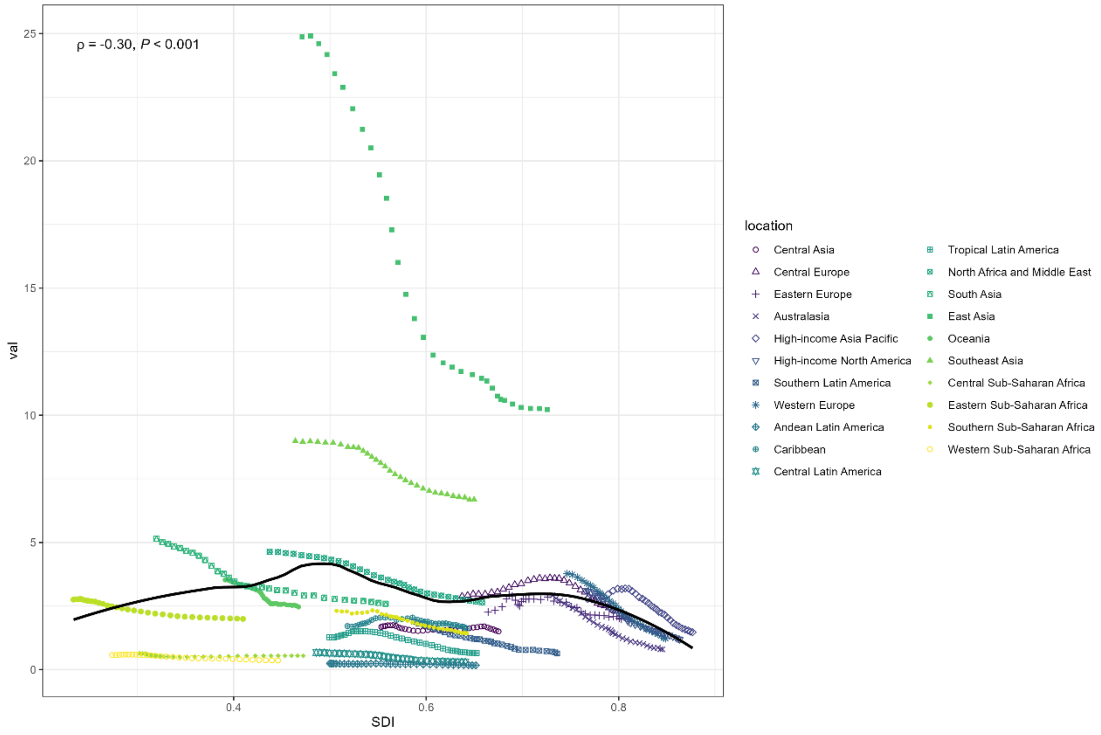

Figure S11. Age-standardized rates of YLLs of NPC attributable to smoking across 21 GBD regions by socio-demographic index from 1990 to 2021.

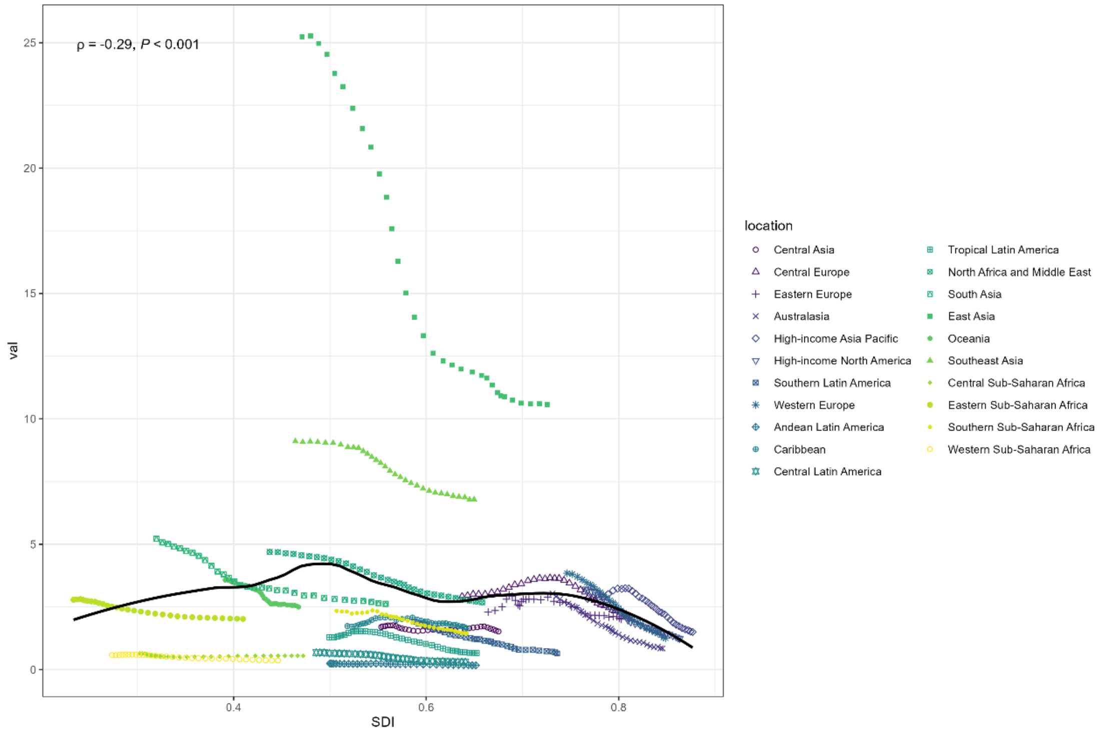

Figure S12. Age-standardized rates of DAYLs of NPC attributable to smoking across 21 GBD regions by socio-demographic index from 1990 to 2021.

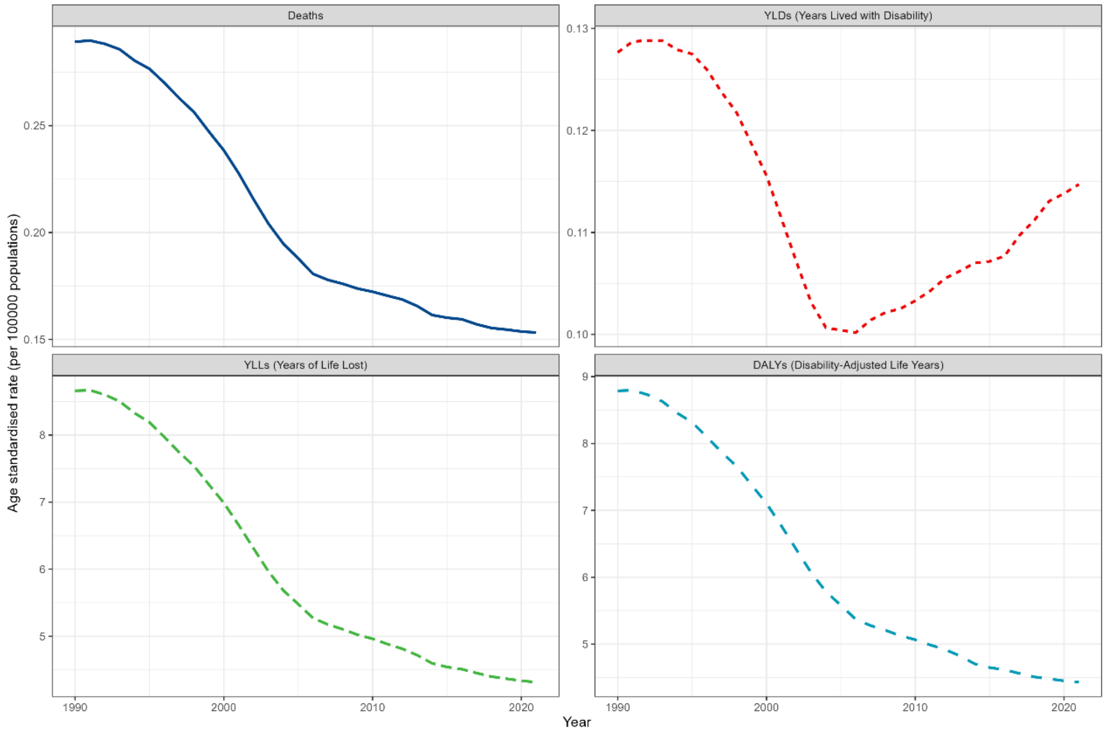

Figure S13. The global changes in Death, YLDs, YLLs, and DALYs from 1990 to 2021.

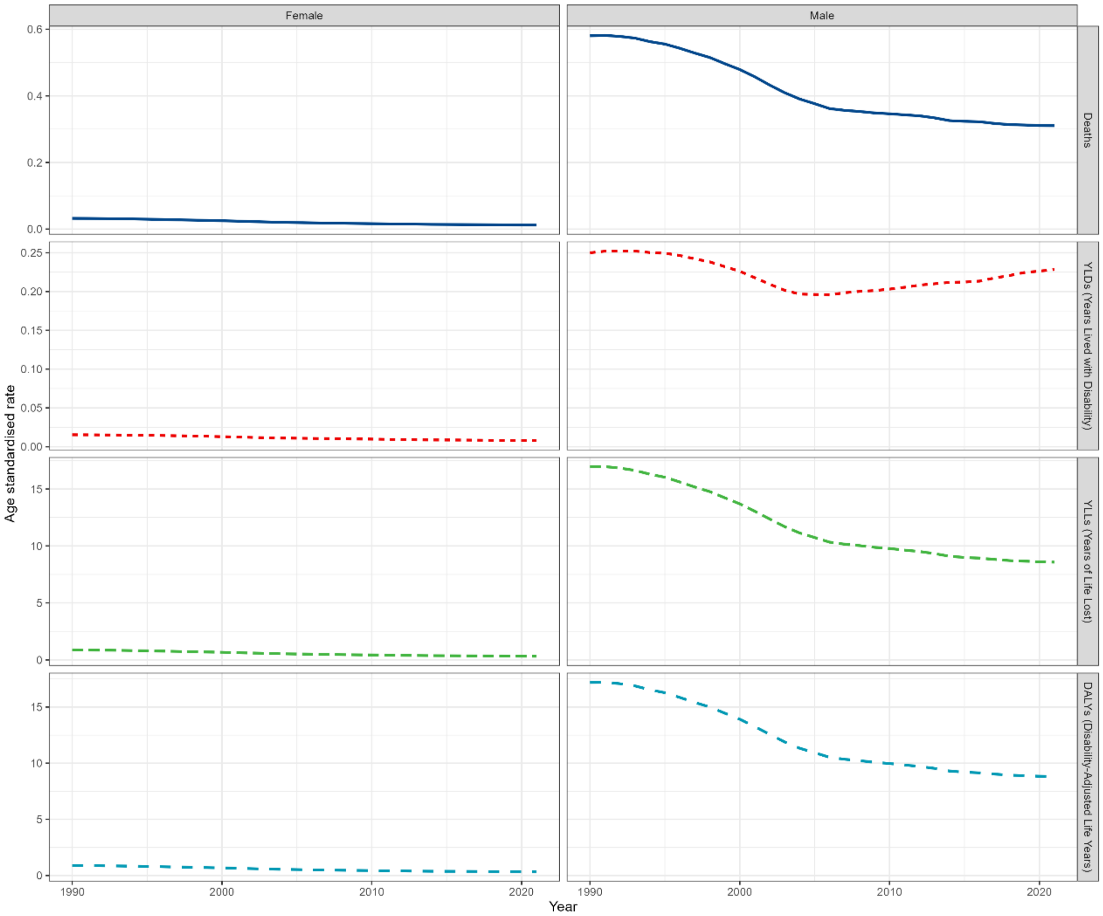

Figure S14. The global changes in mortality, YLDs, YLLs, and DALYs from 1990 to 2021 stratified by gender.

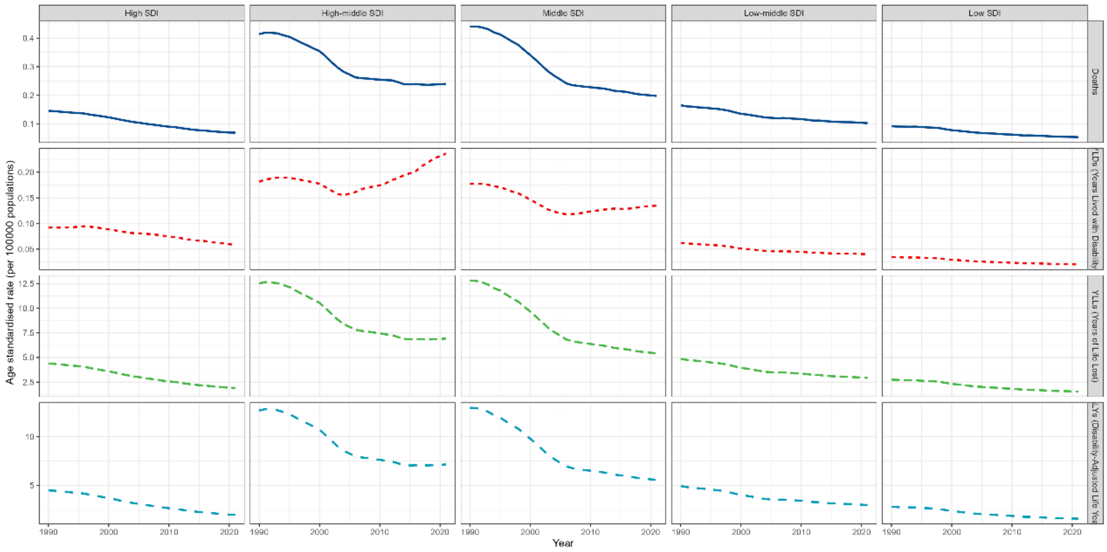

Figure S15. The global changes in mortality, YLDs, YLLs, and DALYs from 1990 to 2021, stratified by Sociodemographic Index.

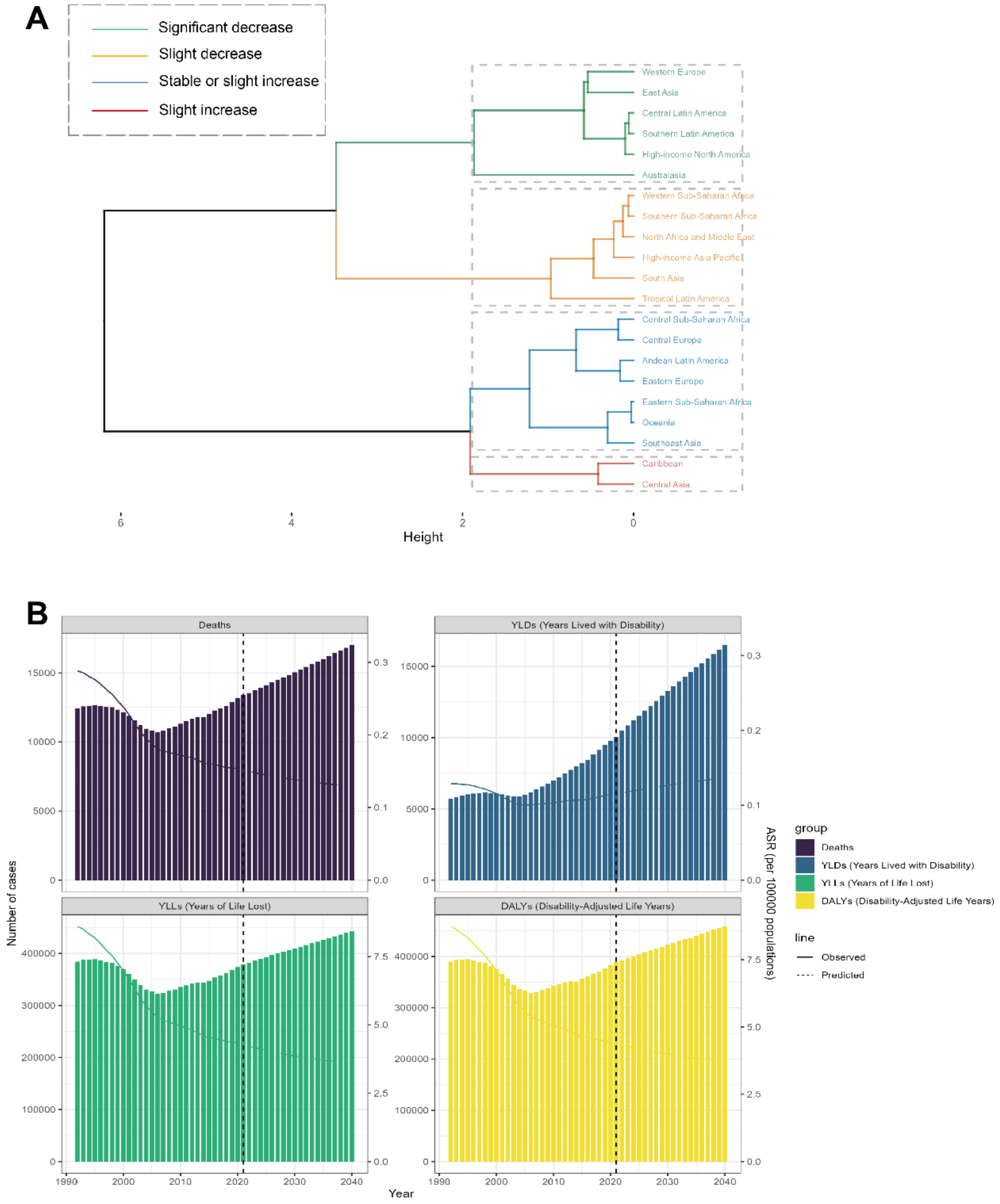

Figure S16. Cluster analysis based on the EAPC of death from 1990 to 2021 (A) and the prediction to 2040 using NORDPRED age-period-cohort model (B). The bar chart represents the number of cases, and lines represent their age-standardized rates. The observed values are on the left side of the black dotted line, and the predicted values after 2021 are on the right side.

© 2025 Liu D. et al.
